# The influence of sleep disorders on perioperative neurocognitive disorders among the elderly: A narrative review

**DOI:** 10.1002/ibra.12167

**Published:** 2024-06-08

**Authors:** Chao Chen, Rui‐Xue Zhai, Xin Lan, Sheng‐Feng Yang, Si‐Jie Tang, Xing‐Long Xiong, Yu‐Xin He, Jing‐Fang Lin, Jia‐Rong Feng, Dong‐Xu Chen, Jing Shi

**Affiliations:** ^1^ Department of Anesthesiology/Department of Neurosurgery The Affiliated Hospital of Guizhou Medical University Guiyang China; ^2^ Department of Gastroenterology and Hepatology The First Affiliated Hospital of Soochow University Suzhou China; ^3^ Department of Anesthesiology, Fujian Provincial Hospital Sheng Li Clinical Medical College of Fujian Medical University Fuzhou China; ^4^ Khoury College of Computer Sciences Northeastern University Boston America; ^5^ Department of Anesthesiology, West China Second Hospital Sichuan University Chengdu China

**Keywords:** insomnia, perioperative neurocognitive disorders, postoperative cognitive dysfunction, postoperative delirium, sleep disorders

## Abstract

This review comprehensively assesses the epidemiology, interaction, and impact on patient outcomes of perioperative sleep disorders (SD) and perioperative neurocognitive disorders (PND) in the elderly. The incidence of SD and PND during the perioperative period in older adults is alarmingly high, with SD significantly contributing to the occurrence of postoperative delirium. However, the clinical evidence linking SD to PND remains insufficient, despite substantial preclinical data. Therefore, this study focuses on the underlying mechanisms between SD and PND, underscoring that potential mechanisms driving SD‐induced PND include uncontrolled central nervous inflammation, blood–brain barrier disruption, circadian rhythm disturbances, glial cell dysfunction, neuronal and synaptic abnormalities, impaired central metabolic waste clearance, gut microbiome dysbiosis, hippocampal oxidative stress, and altered brain network connectivity. Additionally, the review also evaluates the effectiveness of various sleep interventions, both pharmacological and nonpharmacological, in mitigating PND. Strategies such as earplugs, eye masks, restoring circadian rhythms, physical exercise, noninvasive brain stimulation, dexmedetomidine, and melatonin receptor agonists have shown efficacy in reducing PND incidence. The impact of other sleep‐improvement drugs (e.g., orexin receptor antagonists) and methods (e.g., cognitive‐behavioral therapy for insomnia) on PND is still unclear. However, certain drugs used for treating SD (e.g., antidepressants and first‐generation antihistamines) may potentially aggravate PND. By providing valuable insights and references, this review aimed to enhance the understanding and management of PND in older adults based on SD.

## Graphical abstract

This review examines sleep disorders (SD) and perioperative neurocognitive disorders (PND) in the elderly, focusing on their prevalence and interrelation. It highlights SD's role in increasing postoperative delirium, although more evidence is needed regarding its effect on postoperative cognitive dysfunction. Possible mechanisms linking SD to PND include central nervous inflammation, blood–brain barrier disruption, circadian rhythm disturbances, glial cell dysfunction, neuronal and synaptic anomalies, impaired clearance of central metabolic waste, gut microbiome dysbiosis, and oxidative stress. The review also evaluates sleep interventions' impact on PND, including earplugs, eye masks, circadian rhythm restoration, physical exercise, noninvasive brain stimulation, dexmedetomidine, and melatonin receptor agonists, which reduce PND incidence. The influence of other sleep‐improving drugs (e.g., orexin receptor antagonists) and methods (e.g., cognitive behavioral therapy for insomnia) on PND remains uncertain, whereas some SD treatments (e.g., antidepressants and antihistamines) might increase PND risk.

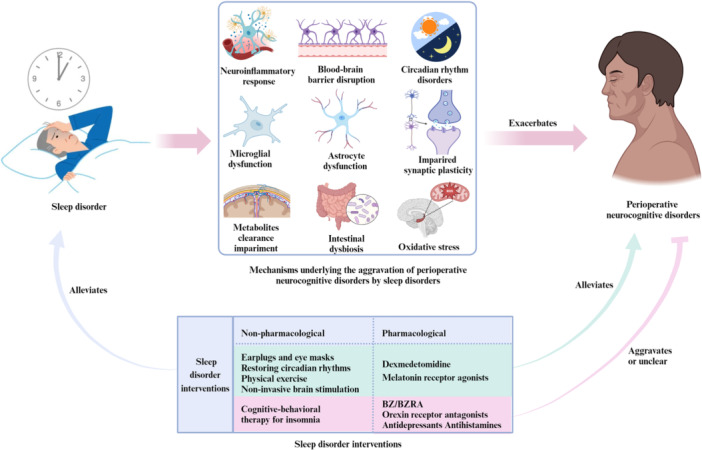

## 1 INTRODUCTION

Perioperative neurocognitive disorders (PND), particularly postoperative delirium (POD) and postoperative cognitive dysfunction (POCD), present significant challenges in elderly surgical patients. These disorders are associated with a spectrum of societal repercussions, including increased postoperative mortality, complications, prolonged hospital stays, higher medical costs, long‐term cognitive decline, reduced functional capacity, early retirement from work, elevated dementia risk, and decreased long‐term survival.^1^ The aging population has led to a growing number of elderly individuals undergoing surgery, making PND a global public health concern. Concurrently, sleep disorders (SD) become a growing threat to the health of the elderly with aging.^2^, ^3^, ^4^, ^5^ SD is not only linked to a range of medical conditions such as diabetes, hypertension, cardiovascular diseases, cancer, metabolic syndrome, and hyperlipidemia, but also associated with endocrine imbalance, immune suppression, increased pain sensitivity, cognitive impairment, and psychiatric abnormalities, all of which negatively impact postoperative outcomes and long‐term survival in elderly patients.^3^, ^4^, ^6^ More importantly, SD may occur after surgery, which can directly exert promoting effects on PND given the critical function of sleep in memory consolidation and the negative impact of sleep disturbances on cognitive function. However, the relationship between SD and the development of PND remains unclear. This review provides the current knowledge of SD and PND in elderly patients, discusses the impact of SD on PND, explores potential mechanisms, and assesses the influence of commonly used SD treatment measures on PND, aiming to provide valuable insights into perioperative management.

## 2 PND

Before the introduction of the term “PND” in 2018, anesthesiologists predominantly referred to “POCD” to describe the postsurgery impairments in functions like language memory, visual memory, attention, concentration, language comprehension, and social skills, following anesthesia and surgery. However, POCD was not categorized as a specific disease entity due to the absence of universally accepted clinical diagnostic criteria. To harmonize terminology and diagnostic standards across disciplines, an international multidisciplinary panel of experts advocated for the adoption of the term PND in 2018.^7^ PND encompasses pre‐existing neurocognitive disorders (NCD), acute POD manifesting within 7 days or before discharge, delayed neurocognitive recovery (DNCR) within 30 days after the operation, and POCD that persists from 30 days up to 12 months postoperatively. The fifth edition of the Diagnostic and Statistical Manual of Mental Disorders (DSM‐5) now categorized PND as a specifics subtype of NCD, providing well‐defined diagnostic criteria and a clearer disease classification for conditions previously labeled as POCD.

## 3 SD

SD entail as range of conditions that detrimentally affect sleep quality, resulting to both subjective and/or objective functional impairments, and negatively impact daytime functioning and overall health. The International Classification of SD, Third Edition (ICSD‐3), released by the American Academy of Sleep Medicine (AASM) in 2014, provides a comprehensive categorization of SD, including insomnia disorders, sleep‐related breathing disorders (SRBD), central disorders of hypersomnolence, circadian rhythm sleep‐wake disorders, parasomnias, sleep‐related movement disorders, and other SD.^8^ During the perioperative period, insomnia disorders and SRBD are particularly prominent, with SRBD contributing to cognitive changes through chronic hypoxia and prolonged carbon dioxide accumulation.

This review primarily focuses on examining the impact of SD, particularly insomnia disorders, on PND. However, diagnosing SD, as per ICSD‐3 or DSM‐5 criteria, presents clinical challenges due to the specialized training required in psychiatric disease diagnosis. The complexity of the diagnostic processes and the need for extensive examinations often reduce patient compliance. Alternative approaches, using validated sleep assessment tools like the Richards‐Campbell Sleep Questionnaire, Pittsburgh Sleep Quality Index, Insomnia Severity Index, or portable monitoring devices can provide a general understanding of a patient's sleep condition, though they may not align with standardized diagnostic classifications for SD.

SD prevalence is notably high and increasing globally. For instance, a 2008 survey reported significant percentages of sleep problems in various populations: 56% in the United States, 31% in Western Europe, and 23% in Japan.^5^ In China, over 21% of people aged 15 and above experience SD.^2^ In the perioperative context, SD can be as high as 60%, influenced by factors such as underlying health conditions, environment, and psychological factors.^3^ It is noteworthy that the incidence of postoperative SD is also significant concern. A multicenter cross‐sectional survey identified a postoperative SD incidence of 64.9%.^9^ Further, a longitudinal study followed 264 postoperative lung cancer patients for 1 year, documented persistently high rates of SD, with incidence of 68.5%, 55.4%, 51.3%, and 49.7% of patients at 1, 5, 9, and 12 months, respectively.^10^ Despite these high prevalence rates, SD have not garnered sufficient attention in the medical community. It is estimated that only a third to a half of those suffering from SD seek medical assistance, and a majority remain unaware of the potential risks associated with these disorders.^11^ Ongoing research continues to reveal the detrimental impacts of SD, highlighting a gap in awareness and treatment that necessitates further attention in clinical practice, particularly in the context of perioperative care.

## 4 IMPACT OF SD ON PND

### 4.1 POD

POD refers to a rapid and fluctuating change in attention, consciousness, and cognitive function after surgery.^7^ It commonly observed in older adults following surgical procedures. A systematic review and meta‐analysis reported that the incidence of noncardiac POD in individuals aged 60 and above is approximately 23.8%.^12^ Influential factors for POD include the type and complexity of surgery, anesthesia, patient health status, and the perioperative environment.^13^ Data from the National Surgical Quality Improvement Program of the American College of Surgeons,^14^ the incidence of POD in older adults is 13.7% after cardiothoracic surgery, 13.0% after orthopedic surgery, 13.0% after general surgery, 11.4% after vascular surgery, 8.0% after neurosurgery, 7.1% after otolaryngology, 6.6% after urology, and 4.7% after gynecological surgery. Other studies have reported rates of delirium after hip fracture surgery (16.9%)^15^ and emergency surgery (26%).^16^ POD is associated with various adverse outcomes, including prolonged hospitalization,^17^ increased perioperative mortality,^18^ increased postoperative complications,^18^ more frequent unplanned intensive care unit (ICU) admissions,^18^ elevated rehospitalization rates,^17^, ^19^ increased medical costs,^20^ as well as short‐term and long‐term cognitive decline.^19^, ^21^ It is also linked to reduced quality of life and an increased risk of dementia.^19^, ^22^ Despite extensive research, preventing and managing POD effectively remains challenging.

SD, present either before or after surgery, are significant risk factors for POD in elderly patients.^13^ Research showed a correlation between reduced slow‐wave sleep presurgery and increased POD risk following cardiac valve replacement surgery.^23^ Likewise, large‐scale study involving 321,818 individuals from the UK Biobank identified an elevated occurrence of POD during hospitalization among patients with SD. Importantly, this risk increased with the severity of SD following surgery.^24^ Subsequent systematic reviews and meta‐analyses have re‐affirmed the association between both preoperative and postoperative SD and an increased risk of POD, even after adjusting for confounders like age and surgical type.^25^, ^26^

Furthermore, POD adversely impacts sleep quality. In ICU settings, delirious patients have been found to experience a significant reduction in the duration of rapid‐eye‐movement (REM) sleep,^27^ and the duration of POD correlated with an increased risk of SD after discharge.^28^ This suggests a vicious cycle where SD contribute to POD development, and in turn, POD exacerbates SD severity. Given this critical connection, sleep interventions have become a significant focus in medical research and practice for addressing POD.^29^

### 4.2 POCD

DNCR and POCD involve a decline in cognitive domains such as complex attention, executive function, learning and memory, language, perceptual‐motor, or social cognition, compared to preoperative levels. DNCR denotes cognitive impairments lasting up to 30 days after surgery, while POCD refers to cognitive impairment occurring between 30 days and 12 months after surgery.^7^ The primary difference between these two lies in their duration: DNCR typically involves temporary cognitive decline with an expected return to normal cognition whereas POCD can persist long‐term and significantly impact patients' lives. Factors influencing the occurrence of POCD include the patient's health status, the type and duration of surgery, intraoperative management, and anesthesia.^1^

In the early postoperative periods (at discharge or 7 days postoperatively) following major noncardiac surgeries, the incidence of POCD ranges from 25.8% to 41.4%, decreasing to 9.9% to 12.7% at 3 months postoperatively.^30^, ^31^ POCD incidences are generally higher in cardiac surgeries, varying widely between 9% and 54%.^32^ However, the use of cardiopulmonary bypass does not appear to have a significant impact on the incidence of POCD after cardiac surgery.^33^ POCD is associated with increased postoperative mortality,^1^ delayed hospitalization,^1^ increased medical costs,^34^ decreased activities of daily living,^1^ early exit from the labor market, decreased functional abilities (such as handling finances and shopping),^35^ decreased long‐term quality of life,^34^, ^36^ and increased long‐term mortality.^30^

Sleep plays a crucial role in memory consolidation, storage, and processing. Aging is associated with changes in sleep patterns and can contribute to the mild cognitive impairment (MCI)^37^ and dementia.^38^ Increasing sleep duration by an hour in older adults with less than 7 h of sleep significantly lowers mild memory‐related cognitive impairment.^39^ Conversely, excessively long sleep duration (more than 10 h per day) is associated with a higher incidence of MCI.^37^, ^40^ MCI serves as a precursor to dementia, with approximately 30%–50% of diagnosed MCI patients progressing to dementia within 5–10 years.

Current clinical evidence on SD's impact on POCD is limited. Observational study revealed an association between preoperative SD and increased DNCR.^41^ Sleeping less than 5 h the night before surgery was linked to postoperative cognitive decline.^42^ Animal models suggested that sleep deprivation increased the POCD occurrence through inflammation activation, blood–brain barrier (BBB) disruption, and microglia and astrocytes activation.^43^ However, large‐scale, multicenter clinical studies are needed to elucidate the impact of SD on POCD.

## 5 THE POTENTIAL MECHANISMS DRIVING SD‐INDUCED PND

**Figure 1:**
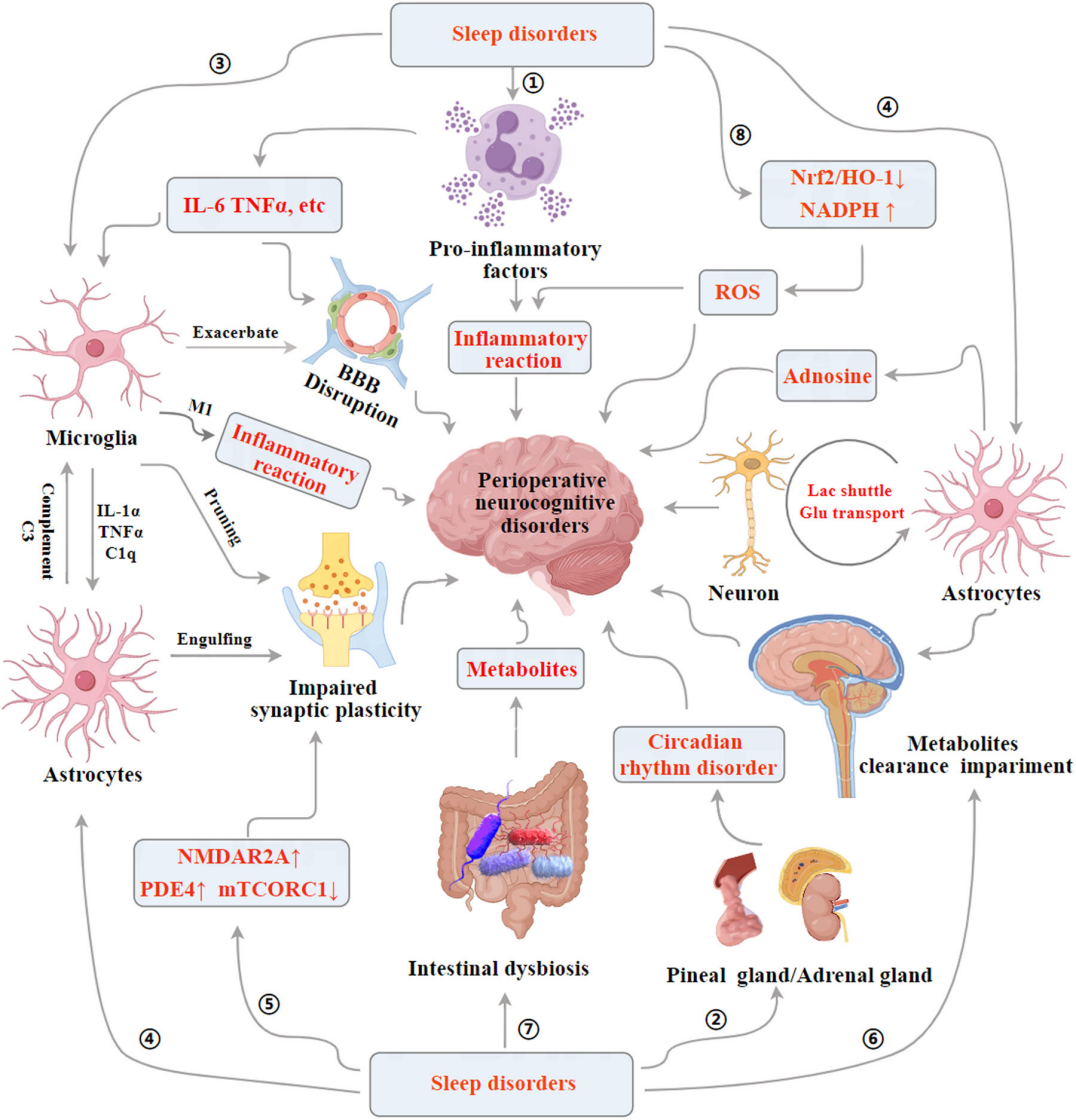
Potential mechanisms underlying the promotion of perioperative neurocognitive disorders by sleep disorders. BBB, blood–brain barrier; HO‐1, heme oxygenase‐1; M1, M1 polarization; mTORC1, mammalian target of rapamycin complex 1; NADPH, nicotinamide adenine dinucleotide phosphate hydrogen; NMDAR2A, NMDAN‐methyl‐D‐aspartate receptor 2A subunit; Nrf2, nuclear factor erythroid 2‐related factor 2; PDE4, phosphodiesterase 4; ROS, reactive oxygen species. [Color figure can be viewed at wileyonlinelibrary.com]

SD potentiate the development of PND via intricate pathways with involvement of following key mechanisms (Figure 1): (1) Enhanced central nervous system (CNS) inflammation driven by increased release of inflammatory cytokines due to sleep disruption; (2) Disruption of circadian rhythm regulation impacting the pineal gland and adrenal cortex secretions, facilitating the onset of PND; (3) Activation of microglial cells triggering a cascade of events including BBB compromise, synaptic damage, and further inflammation, exacerbating PND progression; (4) Impairment in astrocytic functions such as phagocytosis, adenosine metabolism, lactate shuttling, and glutamate transporter regulation, contributing to PND; (5) Dysregulation of protein activities such as mammalian target of rapamycin complex 1 (mTORC1) and phosphodiesterase 4 (PDE4), inhibiting the synthesis of hippocampus‐associated memory proteins and promoting PND; (6) Compromised brain metabolic waste clearance mechanisms, leading to accumulation of neurotoxic substances; (7) Gut microbiome dysbiosis resulting in the production of metabolites that aggravate PND; (8) An imbalance in hippocampal antioxidative and oxidative mechanisms, leading to increased production of reactive oxygen species (ROS), further promoting PND.

### 5.1 Neuroinflammatory response and BBB disruption

The heightened neuroinflammation caused by anesthesia and surgical procedures plays a crucial role in PND. These interventions induce cellular damage, triggering the release of endogenous damage‐associated molecular pattern factors and pro‐inflammatory cytokines such as tumor necrosis factor alpha (TNF‐α) and interleukin 6 (IL‐6).^43^, ^44^, ^45^ This immune response, involving neutrophils and monocytes, serves to counteract tissue damage and maintain homeostasis but also induces a systemic peripheral inflammatory reaction. Inflammatory factors like TNF‐α and IL‐6 increase the permeability of the BBB, facilitating the inflammatory mediators and immune cells, including macrophages, into infiltrate CNS regions like the hippocampus.^44^, ^45^ This series of events leads to neuroinflammation, causing neuronal loss or dysfunction. Notably, TNF‐α in the CNS activates astrocytic TNF receptor 1, leading to sustained functional modification of hippocampal excitatory synapses and impaired memory function.^46^

Sleep deprivation exacerbates both peripheral and central inflammatory responses.^47^ The compromised BBB allows peripheral inflammation to intensify central inflammation,^46^ while central inflammatory mediators, such as prostaglandin D2, diffuse outward, initiating a systemic inflammatory response that can culminate in multiorgan failure.^47^ Moreover, the complement pathway, a crucial component of inflammatory responses, is activated early in the pathogenesis of PND. In a mouse model of sleep deprivation, it was observed that sleep deprivation activated the hippocampal complement C3 pathway in astrocytes, enhancing microglial function and causing cognitive impairment.^48^

The BBB is essential for protecting the CNS. Increased BBB permeability is associated with a higher incidence of POD.^49^ The BBB consists of peripheral cells, neurons, microglia, astrocytic end‐feet, the basement membrane, and vascular endothelial cells. Vascular endothelial cells maintain BBB integrity, while astrocytic end‐feet form an additional barrier communicate with endothelial cells to preserve BBB integrity.

In early inflammatory responses, microglia migrate to vascular endothelial cells, physically contacting them and expressing the tight junction protein Claudin‐5 to repair the damaged BBB and maintain functional integrity. However, prolonged inflammatory stimulation leads to activated microglia start to phagocytose astrocytic end‐feet, reducing BBB integrity and increasing its permeability, with neurotoxic substances into the CNS and subsequent neural damage.^50^ Sleep restriction not only diminishes endothelial cells in the BBB but also reduces tight junction proteins (claudin‐1, claudin‐5, ZO‐2) and increases cyclooxygenase‐2 levels (a critical enzyme in inflammatory responses),^51^ further increasing BBB permeability.^52^

### 5.2 Disruption of circadian rhythms

The disruption of circadian rhythms plays a dual role in PND,^27^ both as a common clinical manifestation and as a significant contributing factor.^53^ Research in mice with delirium have shown pronounced circadian rhythm disruptions, and disturbances in physiological and genetic circadian rhythms increase susceptibility to delirium.^53^ The clock gene E4 promoter‐binding protein (E4BP4) is crucial for hippocampal long‐term potentiation (LTP) and cognitive function, as it suppresses the ERK1/2 cascade reaction and activation of glial cells. Disruption of circadian rhythms leads to a reduction in the expression of E4BP4 in the hippocampal CA1 region, resulting in cognitive impairment.^53^ Moreover, tau protein deposition in the hippocampus and cortex can alter circadian rhythms. In mouse models, circadian rhythm disruption through the overexpression of human P301L tau led to increased wakefulness and decreased nonrapid eye movement (NREM) and REM sleep episodes,^54^ paralleling circadian rhythm disruptions observed in Alzheimer's disease (AD) patients with tau deposits.

The disturbance of the circadian rhythm in melatonin secretion contributes to the onset of PND. The suprachiasmatic nucleus in the hypothalamus regulates melatonin secretion by the pineal gland, synchronizing circadian rhythms in various bodily tissues. Melatonin's antioxidative, anti‐inflammatory, antiapoptotic, and cholinergic system‐restoring properties are crucial for preserving cognitive function. Studies indicated that melatonin can enhance cognition by clearing β‐amyloid (Aβ) and tau, ameliorating insulin resistance,^55^ restoring circadian rhythms,^56^ and reversing changes in brain and muscle aryl hydrocarbon receptor nuclear translocator‐like 1, circadian locomotor output cycles kaput (Clock), and histone deacetylase 3 (HDAC3) protein levels induced by chronic sleep deprivation, to enhance cognitive function in experimental animals.^57^ Chronic sleep deprivation leads to loss of circadian rhythms and reduced melatonin secretion, impacting synaptic plasticity and cognition.^58^

Circadian disruptions significantly affect cortisol, a hormone with a robust diurnal secretion pattern. Cortisol peaks in the early morning and gradually decrease after the onset of nighttime sleep, and reach their lowest levels a few hours into nocturnal sleep. Its circadian rhythmicity is essential for maintaining normal sleep rhythms and cognitive functions. Stressors such as sleep deprivation or chronic sleep restriction disrupt the circadian rhythm of cortisol in the bloodstream, leading to elevated cortisol levels.^59^ While brief elevations in cortisol contribute to increased stress resilience, sustained high cortisol levels can impair cognitive function.^60^ Research has shown that a 5‐day treatment with dexamethasone, which is a glucocorticoid, induces prolonged activation of glucocorticoid receptors, subsequently disrupting circadian gene regulation in the hippocampus. This disruption occurs through the binding of glucocorticoid response elements in the core clock gene Period 1. The loss of circadian variability in hippocampal LTP and impaired hippocampal memory are associated with this disruption.^61^ Cognitive impairment due to chronic hypercortisolemia was observed in common clinical conditions such as Cushing's syndrome, congenital adrenal hyperplasia, and exogenous glucocorticoid therapy.^60^

### 5.3 Dysregulation of microglial function

Microglia, essential immune cells in the CNS, play a crucial role in monitoring the brain's microenvironment, contributing to synaptic activity, pruning, and remodeling, engaging in synaptic trimming, regulating overactive neurons, and phagocytosing damaged neurons and dysfunctional synapses. Microglia secrete brain‐derived neurotrophic factor, which is important for learning and memory by promoting the formation of learning‐dependent synapses.^62^ Synaptic pruning during sleep is indispensable for establishing stable neuronal circuits that sustain normal cognitive function.^63^ Microglial activity and modification capabilities are heightened during sleep but diminish during wakefulness due to the function of neurotransmitters like norepinephrine.^64^ Sleep deprivation results in dysfunctional microglial phagocytosis and synaptic modification, leading to cognitive decline.^65^

The polarization of microglia M1 or M2 states significant impacts PND. Under normal conditions, microglia possess a conservative neural morphology but transform into a “reactive” phenotype upon stimulated. In the CNS, activated microglia can polarized into a pro‐inflammatory (M1 polarization), producing cytokines, chemokines, and high levels of inducible nitric oxide synthase, leading to neurotoxicity and cognitive impairment.^66^, ^67^ Conversely, exposure to anti‐inflammatory factors drives microglia toward an anti‐inflammatory (M2) phenotype, releasing cell factors with neuroprotective and wound‐healing effects.^66^, ^67^ This polarization of microglia offers a potential neuroprotection strategy, considering their dual role in the nervous system.^67^ Sleep deprivation‐induced neuroinflammation leads to a shift in microglial polarization toward the neurotoxic M1 phenotype, resulting in neuronal damage. Understanding and modulating this polarization could be key to addressing the cognitive impairments associated with sleep deprivation and PND.^68^

### 5.4 Dysfunctional astrocytes

Astrocytes, important cells in the brain, maintain the integrity of the BBB and support normal brain function. When activated, astrocytes can exhibit distinct reactive phenotypes: neurotoxic/pro‐inflammatory (A1) or neuroprotective/anti‐inflammatory (A2). The A1/A2 categorization, though an oversimplification, aids in understanding astrocytic phenotypes responses in CNS disorders.^69^ A1 reactive astrocytes upregulate various genes, including those involved in the complement cascade, and release pro‐inflammatory factors such as IL‐1β, TNF‐α, and NO. Microglia activation through the IL‐1α, TNF‐α, and C1q, which can induce the transformation of astrocytes into the A1 reactive phenotype, leading to diminished synaptic generation, reduced phagocytic capacity, and death of neurons and oligodendrocytes.^70^ Conversely, A2 reactive astrocytes upregulate multiple neurotrophic factors and platelet response, benefiting the CNS.^69^ SD can induce pro‐inflammatory microglia, activating astrocytes into a pro‐inflammatory state and triggering secondary neuroinflammation, ultimately resulting in neuronal death.^66^, ^71^ In addition, astrocytes collaborate with microglia in clearing damaged cellular components. Following neuronal death, astrocytes rapidly polarize and engulf dendritic apoptotic bodies, while microglia phagocytose dendritic tips and cell bodies, maintaining brain health.^72^ SD increase the phagocytic activity of cortical astrocytes and activate microglia, leading to the engulfment of normal synaptic components and cognitiveimpairment.^73^

Astrocytes also modulate cognitive function through neurotransmitter regulation. They release adenosine triphosphate, hydrolyzed into adenosine, a neurotransmitter involved in sleep regulation. However, astrocyte‐derived adenosine has been shown to mediate synaptic alterations and memory deficits in sleep deprivation mice.^74^ Additionally, astrocytic ketone carboxylase converts glucose to glutamate, providing extra glutamate to the brain and facilitating its transport into synapses through glutamate transporter 1 and glutamate aspartate transporter. This process is crucial for glutamate signaling in synaptic plasticity, which underlies memory consolidation.^75^ Furthermore, astrocyte‐generated L‐lactate is essential for the formation of long‐term memory in neurons. Activation of astrocytic β2‐adrenergic receptors stimulates the production of L‐lactate, promoting memory formation.^76^ Short‐term β2 agonists improves memory by increasing astrocytic L‐lactate output. However, prolonged activation of β2‐adrenergic receptors reduces cognitive abilities by decreasing the supply of astrocytic L‐lactate to neurons, leading to internalization of these receptors.^77^ Chronic sleep deprivation induces a physiological state similar to persistent stress, resulting in elevated catecholamine levels.^78^ This prolonged activation of β2‐adrenergic receptors may impact cognitive function by influencing these signaling pathways involved in memory regulation.

### 5.5 Neuronal and synaptic dysfunction in hippocampus

The hippocampus, vital for learning and memory, undergoes synaptic plasticity called LTP, which is crucial for cognitive function. The cyclic adenosine monophosphate (cAMP) pathway, which modulates N‐methyl‐D‐aspartate (NMDA) receptor‐dependent LTP in the hippocampal CA1 region, is pivotal in this process. Sleep deprivation disrupts this balance by upregulating PDE4, an enzyme responsible for cAMP degradation. This leads to reduced cAMP levels, impairing LTP in the hippocampal CA1 region and adversely affect memory function.^79^ Sleep deprivation also alters the expression of NMDA receptors in the hippocampus. Brief sleep restriction increases the NR2A subunit of NMDA receptors, changing the NR2A/NR2B ratio and impacting hippocampal LTP.^80^ While alterations can be reversed with sleep recovery, the long‐term effects of prolonged sleep restriction remain unclear. Additionally, sleep deprivation significantly reduces the expression and phosphorylation levels of glutamate receptors in the posterior hippocampus, weakening glutamate signaling and affecting LTP.^81^ Changes in synaptic plasticity also involve alterations in dendritic spine numbers on neurons. Sleep deprivation enhances the activity of phosphodiesterase‐4A5, impairing cAMP‐protein kinase A‐LIM kinase signaling. This results in an increased expression of cofilin, a protein involved in actin filament dynamics, ultimately reducing the number of dendritic spines in the hippocampal CA1 region.^82^ Other studies have observed decreased levels of postsynaptic density protein 95 and memory impairment.^83^

Protein synthesis is necessary for synaptic plasticity and long‐term memory. The kinase complex mTORC1 regulates protein synthesis by phosphorylating eukaryotic translation initiation factor 4E‐binding protein 2 (4EBP2), inhibiting its activity. Sleep restriction impairs mTORC1‐mediated phosphorylation of 4EBP2, suppressing protein synthesis in the hippocampus and resulting in memory deficits.^84^ Histone acetylation also plays a role in hippocampal protein synthesis. Acute sleep deprivation reduces the expression of acetylated histone markers H3K9 and H4K12, while increasing HDAC2 abundance. Treatment with the histone deacetylase inhibitor suberanilohydroxamic acid (SAHA) restores histone acetylation levels, alleviating the inhibitory effects of sleep deprivation on LTP and long‐term memory.^85^ Inhibiting histone deacetylase activity during chronic sleep deprivation can further enhance long‐term memory consolidation.^86^ Thus, SD may disrupt hippocampal protein synthesis through multiple pathways, leading to cognitive impairments associated with memory.

### 5.6 Impaired clearance of metabolic waste

In PND, the dysregulation of protein clearance, including Aβ, tau, and inflammatory mediators, is a crucial mechanism. The brain relies on the exchange of cerebrospinal fluid (CSF) and interstitial fluid (ISF) through the perivascular space (PVS) to remove neurotoxic molecules associated with neurodegenerative disorders and maintain a healthy brain environment.^87^ This exchange system, known as the cerebral “lymphatic system,” is more effective than the traditional circulation through the ventricles and subarachnoid space.^87^ A key component of this system's functionality is the aquaporin‐4 (AQP‐4) water channels located at the astrocytic endfeet adjacent to the vascular system. AQP‐4 channels facilitate the directional flow of CSF from the PVS into the brain parenchyma.^88^ The regulation of CSF and AQP4 dynamics is influenced by circadian rhythms.^89^ During sleep or anesthesia, the interstitial space expands, significantly enhancing the convective exchange between CSF and ISF. This enhanced exchange more efficiently clears neurotoxic macromolecules, such as Aβ.^90^ However, disruptions in sleep can interfere with these processes, leading to increased concentrations of Aβ and tau proteins within the brain, ultimately contributing to cognitive impairment.^90^, ^91^ This relationship underscores the importance of sleep‐in maintaining brain health and highlights the potential impacts of sleep disruption on the development and progression of NCD, particularly in the perioperative context.

### 5.7 Dysbiosis of gut microbiota

Dysbiosis of the gut microbiota plays a significant role in the context of PND. SD result in significant changes to the gut microbial composition, characterized by an increase in certain microbial families such as NK4A136, Lactobacillus, Streptococcus, and Bifidobacterium. These alterations correlate with shift in serum metabolic products (like aspartic acid, phenylalanine, Phe‐Phe, Asp‐Phe, Phe‐Trp, etc.) and fluctuations in pro‐inflammatory factors (such as IL‐1β, TNF‐α, etc.).^92^ The dysregulation of the gut microbiota triggers the activation of the NOD‐like receptor family pyrin domain containing 3 (NLRP3) inflammasome in the intestines, leading to compromised integrity of the intestinal mucosal barrier and resulting in both local and peripheral inflammatory responses.^93^ Crucially, gut‐derived inflammatory factors can induce inflammation and microglial activation in key cognitive regions like the hippocampus and medial prefrontal cortex through the TLR‐4/NF‐κB pathway, contributing to cognitive impairment.^94^ Strategic interventions targeting the consequences of disrupted microbiota due to sleep deprivation are emerging as promising therapeutic approaches. For instance, administering *Akkermansia muciniphila* has been effective in inhibiting microglial phagocytosis of synapses, thus preventing cognitive impairment in sleep‐deprived mouse models.^95^ Similarly, supplementation with metabolites from Faecalibacterium, such as butyrate salts, shows potential in ameliorating intestinal mucosal damage in sleep‐deprived mouse models.^96^ Notably, clinical trials have demonstrated the impact of perioperative probiotic supplementation on postoperative cognitive function.^97^

These findings highlight the significant interplay between sleep, gut microbiota, and cognitive health, particularly in the context of PND, suggesting that targeting gut microbiota through dietary or probiotic interventions could be a viable strategy to mitigate the adverse cognitive effects associated with SD and perioperative stress.

### 5.8 Oxidative stress

Insufficient sleep leads to oxidative stress, which has widespread effects on various organs, having a significant implication for the brain.^98^ Prolonged sleep deprivation causes an accumulation of ROS within the intestines, proving fatal in experimental animal models.^99^ In hippocampal CA1 neurons, crucial endogenous antioxidants, such as nuclear factor erythroid 2‐related factor 2 (Nrf2) and heme oxygenase‐1 (HO‐1), are downregulated due to sleep deprivation. Activating the Nrf2/HO‐1 pathway is emerging as a promising strategy to counteract cognitive impairments induced by sleep deprivation.^100^ Nicotinamide adenine dinucleotide phosphate hydrogen oxidase (NOX), an enzyme critical in ROS generation, shows increased expression in the hippocampus and cortex following fragmented sleep, resulting in an excess of ROS and promoting the development of POCD.^101^, ^102^

This elevation results in an overproduction of ROS, contributing to the development of POCD. This relationship between oxidative stress, particularly through the Nrf2/HO‐1 and NOX pathways, and cognitive health underscores the importance of adequate sleep and the potential of targeted therapeutic interventions to mitigate the adverse cognitive effects associated with sleep deprivation. Addressing oxidative stress in the context of sleep deprivation could, therefore, be a key component in managing and preventing POCD, especially in perioperative care.

### 5.9 Additional potential mechanisms

Functional magnetic resonance imaging studies have provided insights into how sleep deprivation alters brain network connectivity, with notable implications for cognition. Even brief periods of sleep deprivation, such as a single night, can lead to significant reductions in hippocampal activity, which is crucial for memory encoding. Interestingly, when hippocampal activity, vital for memory function, is compromised, there appears to be a compensatory increase in coupling between the hippocampus and the primary alertness networks located in the brainstem and thalamus.^103^ Additionally, sleep deprivation affects connectivity within various brain networks. It leads to changes in the dorsal attention network, visual network, and default mode network.^104^ These modifications can significantly impact cognitive processes such as attention and memory. Prolonged sleep deprivation further exacerbates these effects, with reduced activation in key areas like the prefrontal cortex, posterior parietal cortex, and supplementary motor area during working memory tasks.^105^ While the exact nature of these changes – whether they are adaptive responses by the brain to cope with the lack of sleep or direct impairments caused by sleep deprivation – remains unclear, their significant impacts on cognition are evident.

These findings highlight the complex relationship between sleep, brain network functionality, and cognitive performance. Understanding these relationships is crucial for developing strategies to mitigate the cognitive deficits associated with sleep deprivation.

## 6 INTERVENTIONS TARGETING SD AND THEIR IMPACT ON PND

### 6.1 Nonpharmacological interventions

#### 6.1.1 Earplugs and eye masks

Earplugs and eye masks are simple and cost‐effective ways to improve sleep, especially in places like ICUs where sleeping can be hard. Studies show that patients in the postanesthesia care unit sleep better on their first night after surgery when they use earplugs and eye masks.^106^ Using polysomnography, the best method for checking sleep, one study found that earplugs and eye masks helped critically ill patients sleep less fitfully and have more deep sleep (N3 sleep).^107^ Moreover, these simple interventions have shown potential preventing delirium. Research suggests that coronary patients in the ICU who used earplugs and eye masks at night experienced a reduced incidence of delirium.^108^ A meta‐analysis involving 1445 subjects indicated that using earplugs alone in the ICU can decrease the risk of delirium.^109^ Another larger meta‐analysis corroborated the benefit of earplugs and eye masks on sleep quality and delirium prevention in ICU patients.^110^ However, the direct impact of earplugs and eye masks on cognitive function has not been extensively researched. Given the disruptive influence of light and noise on sleep, patients, particularly those in noisy environments like ICUs, might benefit from using earplugs and eye masks during sleep. These interventions offer a straightforward and noninvasive approach to enhance sleep quality, which is crucial for recovery and overall well‐being in the postoperative period.

#### 6.1.2 Restoring circadian rhythms

Disruption of the circadian sleep rhythm significant contributes to the onset of delirium and dementia in the elderly population. A comprehensive long‐term study involving 53,417 patients established a strong association between circadian rhythm disruption in middle‐aged and elderly individuals and an increased incidence of delirium and dementia.^111^ Restoring circadian rhythms is a multifaceted process, encompassing interventions like aligning light exposure with the circadian rhythm, minimizing unnecessary sleep interruptions at night, managing pain effectively, reducing noise levels, providing informational support, and encouraging wakefulness during the daytime hours. These strategies are especially crucial in mitigating PND.^112^ The importance of these interventions is particularly pronounced in ICU settings, where disruptions to the sleep‐wake cycle are common.^29^ Reviews and analyses of lots of studies show that helping ICU patients keep a regular sleep rhythm, or using many different nondrug methods to fix sleep patterns, can make sleep better and lower the chances of delirium.^113^ Similar evidence from non‐ICU settings reinforces that the effectiveness of optimizing sleep rhythms can effectively decrease the occurrence of delirium.^114^ Both pharmacological and nonpharmacological interventions targeting sleep and circadian rhythms are becoming increasingly recognized as vital components in the prevention of PND. These approaches underline the importance of holistic patient care, considering not only the immediate physical health needs but also the broader aspects of patient well‐being, such as sleep quality and mental health. By addressing these factors, healthcare providers can contribute significantly to the prevention and management of delirium and dementia, particularly in vulnerable populations like the elderly.

#### 6.1.3 Cognitive‐behavioral therapy for insomnia (CBT‐I)

CBT‐I is the preferred nonpharmacological treatment for insomnia and is recommended for insomnia stemming from various causes.^4^ Studies have shown that CBT‐I can improve cognitive function in individuals with comorbid MCI and insomnia,^115^ as well as in chronic insomnia patients.^116^ However, the benefits of CBT‐I on cognitive function are not uniformly observed across all studies.^117^ Notably, in patients with coexisting sleep apnea, treatments incorporating CBT‐I may lead to cognitive impairment, potentially due to the further reduction in sleep time as a result of the sleep restriction component of CBT‐I.^118^ Given the limited and mixed evidence regarding the impact of CBT‐I on cognition, more research is required to gain a clearer understanding of its effects. Currently, there is no research specifically addressing the impact of CBT‐I on delirium. Considering the time required for learning and the onset of effects of CBT‐I therapy, and even assuming good patient compliance, initiating CBT‐I briefly during the perioperative period is unlikely to have a significant impact on PND. This suggests that while CBT‐I is a valuable tool for treating insomnia and potentially improving cognitive function in some cases, its effectiveness in mitigating PND, especially when introduced in the short term during the perioperative period, may be limited. Therefore, a comprehensive approach, possibly combining both pharmacological and nonpharmacological strategies, may be more effective in managing insomnia and its cognitive consequences in the perioperative setting.

#### 6.1.4 Physical exercise

Physical exercise is highly recommended as a nondrug way to deal with sleep problems.^4^ Beyond its established benefits for sleep, physical exercise also provides cognitive benefits through various mechanisms, including enhanced synaptic plasticity, improved mitochondrial function, increased cytokine release, improved brain metabolism, and modulation of the gut microbiota.^119^ The positive effects of physical exercise extend to PND. For example, regular exercise has been shown to remarkable reduction in the incidence of POD in individuals aged 60 and above undergoing elective orthopedic surgery.^120^ Additionally, a 12‐week early moderate‐intensity resistance training program resulted in cognitive recovery improvements in patients following cardiac surgery.^121^ Supporting these findings, a 12‐week Tai Chi practice not only reduced peripheral blood IL‐6 levels but also improved cognitive function in older adults.^122^ Animal studies further underscore the preventive potential of physical exercise against cognitive impairment and anxiety‐like behaviors induced by sleep deprivation, acting through multiple pathways.^123^ These findings highlight the multifaceted benefits of physical exercise, particularly during the perioperative period for elderly patients. The advantages extend beyond cognitive health, including mitigating pulmonary complications, enhancing gastrointestinal function, and reducing the risk of lower limb venous thrombosis.

Given these broad positive impacts, it is advisable for healthcare providers to develop personalized exercise regimens for elderly patients, particularly in the perioperative context. These regimens should be tailored to individual patient conditions, taking into account their specific health status, surgical procedures, and overall recovery goals. By integrating physical exercise into perioperative care plans, patients can potentially experience enhanced recovery outcomes, including better cognitive function and overall well‐being.

#### 6.1.5 Noninvasive brain stimulation (NIBS)

NIBS, such as repetitive transcranial magnetic stimulation (rTMS) and transcranial electric stimulation (TES), is gaining interest as a potential and cost‐effective approach for managing sleep issues.^4^ The underlying principle involves targeted stimulation of specific neuronal populations in regions like the left dorsolateral prefrontal cortex (DLPFC), temporoparietal area, and primary motor cortex to modulate brain activity and influence sleep. While comprehensive evidence from multicenter, large‐sample randomized controlled trials (RCTs) is still emerging, existing studies have shown significant potential for NIBS in treating insomnia.^124^, ^125^

Transcranial direct current stimulation (tDCS), a form of TES, has shown improvements age‐related memory declines^126^ and reduced reaction times in cognitive tasks.^127^ Systematic reviews and meta‐analyses indicate that tDCS treatment in the elderly can slow down the progression of cognitive decline^128^ and enhance performance in various cognitive and memory tasks.^129^ These benefits of extend to patients with MCI and AD.^130^ A recent study by Tao et al.^131^ reported that anodal tDCS over the left DLPFC reduced the incidence of POD in elderly patients undergoing lower limb arthroplasty.

rTMS is widely researched for its impact on cognitive function. Meta‐analysis found that rTMS targeting the DLPFC could enhance cognitive function in patients with age‐related neurodegenerative diseases.^132^ High‐frequency rTMS over the left DLPFC and low‐frequency rTMS over the right DLPFC have been found to improve memory function in patients with MCI and AD, while high‐frequency rTMS over the right inferior frontal gyrus enhanced executive function.^133^ Different brain regions targeted by rTMS can produce varying effects. A study evaluating the efficacy of rTMS on delirium showed that a single session of left DLPFC rTMS could temporarily alleviate the severity of delirium but did not reduce the incidence of delirium 3 days after treatment.^134^ The beneficial effects of rTMS treatment are also evident in improving episodic memory in individuals with cognitive impairment^135^ and restoring cognitive impairment in patients with celiac disease.^136^ Currently, no reports are available regarding the preventive effects of rTMS on delirium, emphasizing the need for additional research in this area.

In summary, NIBS techniques like tDCS and rTMS offer promising options for treating SD and enhancing cognitive function, potentially useful in perioperative care. However, more research is necessary to fully understand their effectiveness and best use in these settings.

### 6.2 Pharmacological interventions

#### 6.2.1 Dexmedetomidine

Dexmedetomidine, a selective alpha‐2 adrenergic agonist, has emerged as an effective agent in managing SD and PND. For elderly noncardiac surgical patients in the ICU, low‐dose dexmedetomidine (0.1 µg/kg/h for 15 h) significantly improves sleep quality. It enhances NREM N2 sleep, total sleep duration, sleep efficiency, and subjective sleep quality.^137^ A retrospective analysis of major noncardiac surgical procedures indicated that intraoperative administration of low‐dose dexmedetomidine (0.2–0.4 µg/kg/h) reduced the incidence of severe postoperative SD.^138^ Consistent findings from meta‐analyses suggest that both intraoperative and postoperative use of dexmedetomidine improves patient‐reported sleep quality and objective sleep measures.^139^, ^140^ However, the impact of preoperative dexmedetomidine on patient sleep quality and its relationship with PND remain uncertain.

The impact of dexmedetomidine on postoperative PND has been extensively studied, particularly in relation to its effect on POD. Dexmedetomidine administered during the postoperative ICU sedation has been shown to reduce POD risk compared to propofol.^141^ A meta‐analysis involving 1387 patients further supported these findings, showing a decreased incidence of POD following perioperative dexmedetomidine administration in postcardiac surgery patients.^142^ However, another meta‐analysis involving 4090 patients did not yield statistically significant differences.^143^ In noncardiac surgery, a recent systematic review with a substantial sample size (4015 cases) concluded that perioperative dexmedetomidine had a favorable effect on POD in elderly patients (>65 years) undergoing major noncardiac surgeries, whereas its impact on individuals under 65 years of age remains uncertain.^144^ The effect of dexmedetomidine on POCD remains a topic of ongoing research.

In summary, dexmedetomidine shows promise in improving sleep quality and reducing the incidence of POD in specific patient populations, particularly the elderly. Its effects on POCD and younger patients require further investigation to establish clear clinical guidelines.

#### 6.2.2 Benzodiazepines (BZ) and benzodiazepine receptor agonists (BZRA)

BZ and BZRA are commonly prescribed for the short‐term treatment of insomnia, often for periods of less than 4 weeks. They are used either in conjunction with CBT‐I or as a standalone treatment. While short‐term use is generally considered relatively safe, there are concerns about their impact on cognitive function, particularly in the elderly and in perioperative settings.^4^ For instance, a randomized trial with elderly patients indicated that intraoperative use of midazolam, which is classified as a BZ drug, increased the incidence of DNCR at 7 days postsurgery, but no cognitive differences were noted at 1 year.^145^ The long‐term impact of BZ on cognitive function remains contentious. Recent meta‐analyses suggest that long‐term BZ use does not necessarily lead to cognitive decline in the general adult population.^146^ However, in elderly populations, prolonged exposure has been associated with an increased risk of dementia and AD.^147^ Considering the various side effects of BZ, such as tolerance, dependence, nocturnal confusion, falls, and hangover effects,^4^ their long‐term use should be approached with caution.

Perioperative exposure to BZ has been linked to POD in elderly noncardiac surgical patients. Consequently, guidelines have recommended avoiding routine preoperative use of BZ to mitigate the risk of POD.^112^ However, a recent systematic review and meta‐analysis indicated that intraoperative use of BZ did not increase the risk of POD or compromise intraoperative awareness, and it actually reduced intraoperative awareness.^148^ These findings suggest a need to re‐evaluate the association between perioperative benzodiazepine use and POD.

Based on the currently limited research evidence, BZRA have a relatively minor effect on cognition. Studies have not found a significant cognition impact of BZRA on patients with insomnia.^149^ However, abrupt discontinuation of long‐term BZRA use may lead to withdrawal delirium.^150^ Additionally, preoperative long‐term use of BZRA has been associated with an increased risk of POD in hip joint surgery.^151^

In summary, while BZ and BZRA can be effective for short‐term management of insomnia, their potential cognitive side effects, particularly in the elderly and in the perioperative period, necessitate careful consideration and judicious use. Further research is essential to clarify their long‐term impact on cognitive health and their relationship with PND.

#### 6.2.3 Melatonin receptor agonists

Melatonin receptor agonists, such as ramelteon, are recommended in European and American guidelines for managing insomnia, although the efficacy of melatonin in treating chronic insomnia is considered limited. The potential impact of melatonin on PND has garnered interest, but the evidence remains mixed.^4^ A study involving elderly patients scheduled for hip replacement surgery found that oral administration of 1 mg of melatonin 1 h before bedtime for five consecutive nights, starting from the night before surgery, improved early postoperative cognitive function.^152^ However, the clinical evidence supporting melatonin's effect on cognitive function remains insufficient, despite animal experiments suggesting potential cognitive benefits such as regulating circadian rhythm disruption, clearing free radicals, and exerting antioxidative, anti‐inflammatory, and immune modulatory effects.^153^

The effects of melatonin and its receptor agonists on delirium are still debated. A RCT involving elderly patients (≥65 years) undergoing elective surgery under general anesthesia, receiving oral administration of 8 mg of ramelteon every night from the night before surgery until the 5th postoperative day, did not observe a reduced incidence of POD compared to the placebo group.^154^ Similar findings were reported in orthopedic surgery.^155^ However, a systematic review and meta‐analysis indicated that melatonin receptor agonists have significant preventive effects on delirium but did not demonstrate therapeutic effects.^156^

Given the variability in study populations, assessment methodologies, drug regimens, and the resulting diverse study outcomes, further investigation is required to clarify the impact of melatonin receptor agonists on PND. The research is crucial to understand better the role of melatonin in perioperative care, particularly regarding cognitive function and delirium prevention in elderly patients undergoing surgery.

#### 6.2.4 Dual orexin receptor antagonists (DORA)

DORA represent a relatively new class of medications used for treating insomnia, and they have shown promise due to their minimal impact on cognitive function at therapeutic doses. This characteristic is particularly advantageous for insomnia patients with pre‐existing cognitive impairment.^157^ While animal experiments largely point to the cognitive advantages of DORA, the clinical evidence is still inconclusive. Two DORA, daridorexant and suvorexant, have been recommended in guidelines for the treatment of insomnia.^4^ Research by Lucey et al. found that suvorexant rapidly reduced tau phosphorylation and amyloid‐beta concentrations in the CNS of patients with cognitive impairment. However, the study did not assess whether this reduction led to improved patient cognition.^158^ Retrospective analyses have also shed light on that suvorexant and lemborexant (a DORA) reduced the incidence of delirium in ICU patients.^159^, ^160^ Additionally, a prospective study observed the preventive effect of suvorexant on delirium in elderly patients admitted to the emergency department.^161^

Given their relatively recent introduction, DORA require further research to fully understand their impact on cognitive function and delirium, particularly in perioperative and critical care settings. Future studies should investigate long‐term cognitive outcomes and explore the potential therapeutic benefits of DORA in various patient populations, especially those at risk for cognitive impairment or delirium.

#### 6.2.5 Antidepressants and antihistamines

Antidepressants, including low‐dose sedating agents like agomelatine, amitriptyline, doxepin, mianserin, mirtazapine, trazodone, trimipramine, and others, are commonly prescribed for elderly patients with insomnia, especially when comorbid depression is present.^4^ Cognitive impairment, commonly seen in moderate to severe depression, is an important indicator of antidepressant effectiveness. Some studies have shown that standard antidepressant therapies can improve cognitive function like language memory, working memory, and reaction time in depressed patients.^162^ However, a systematic review suggests mixed outcomes, with some studies indicating no significant cognitive effects or even potential memory function harm.^163^ In perioperative settings, antidepressant use has been linked to increased POD risk in elderly patients undergoing major joint surgeries, suggesting a potential contribution to the occurrence of PND.^164^

#### 6.2.6 Antihistamines

First‐generation antihistamines like diphenhydramine and promethazine, known for their sedative properties, have been used to treat SD due to their ability to cross the BBB. These agents have historically been used in the treatment of SD.^4^ However, the utilization of first‐generation antihistamines in SD management is currently limited. These drugs have a significant impact on cognitive function, with long‐term use of diphenhydramine associated with cognitive impairment in the elderly population. Due to its prolonged duration of action, even when taken the night before, it can disrupt daytime cognitive performance and overall safety.^165^ Both diphenhydramine and promethazine exposure have been linked to an increased risk of delirium in elderly patients during hospitalization.^166^ Consequently, it is recommended to avoid first‐generation antihistamines during the perioperative period, considering alternative medications for addressing SD.

## 7 DISCUSSION AND CONCLUSION

The association between SD in elderly individuals and the development of PND is well‐established, though challenged by diagnostic variability and comorbidities. Future research should focus on standardizing diagnostic protocols, employing wearable devices for objective sleep data, and utilizing longitudinal studies with advanced neuroimaging.

**Figure 2:**
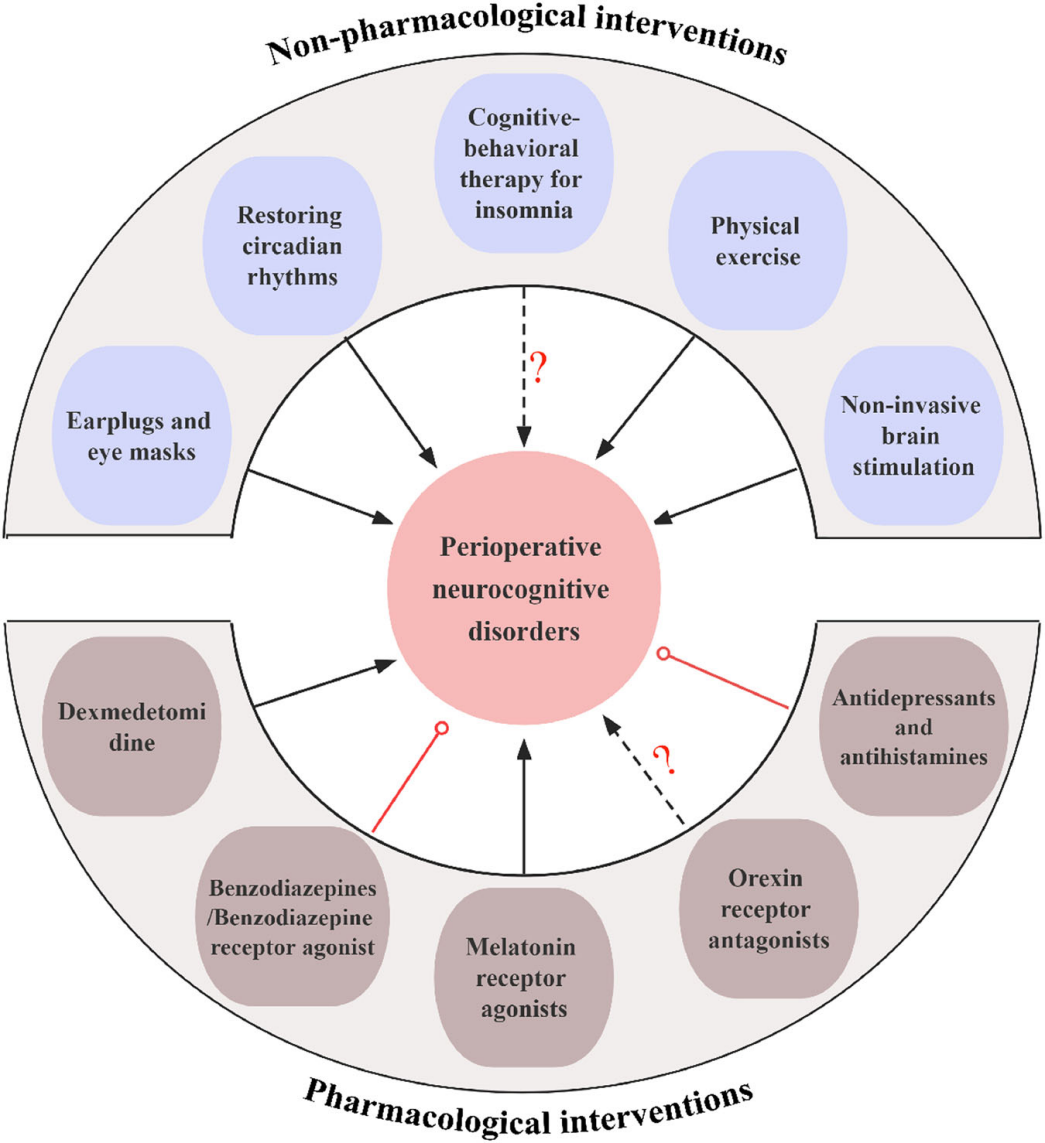
Impact of common sleep interventions on PND. Earplugs and eye masks, restoration of circadian rhythm, physical exercise, noninvasive brain stimulation, dexmedetomidine, and melatonin receptor agonists are associated with a decreased occurrence of PND. The effects of cognitive behavioral therapy for insomnia and orexin receptor agonists on PND remain uncertain. Benzodiazepines and benzodiazepine receptor agonists, along with antidepressants and antihistamines have the potential to promote the development of PND. PND, perioperative neurocognitive disorders. [Color figure can be viewed at wileyonlinelibrary.com]

Multiple mechanisms link SD to PND (Figure 1), including neuroinflammatory responses, BBB permeability, microglia and astrocyte functioning, synaptic plasticity alterations, neurotransmitter imbalances, protein synthesis impairments, brain waste clearance abnormalities, hippocampal oxidative stress, changes in gut microbiota and metabolites, and connectivity abnormalities within brain networks. Clinical settings have identified various multicomponent interventions, both pharmacological and nonpharmacological, to improve sleep quality and mitigate PND (Figure 2). These include eye masks, earplugs, circadian rhythm restoration, physical exercise, cognitive‐behavioral therapy, transcranial electrical stimulation, and medications like dexmedetomidine, melatonin receptor agonists, and DORA. Caution is advised with medications like BZ, antidepressants, and first‐generation antihistamines due to potential adverse effects on PND. Future approaches should focus on personalized and interdisciplinary strategies to enhance postoperative cognitive‐related outcomes in the elderly.

### 7.1 Future perspectives

First, while the detrimental effects of SD on cognitive function in the elderly are established, clinical evidence regarding their impact on postoperative cognition, particularly long‐term outcomes, is still insufficient, necessitating further investigation. Second, the mechanisms through which SD contribute to PND are complex. Although some mechanisms have been identified, their clinical translation remains challenging, and exploring additional mechanisms could offer more options for clinical applications. Third, dexmedetomidine presents as a promising option for managing SD and PND in the perioperative context, with its nasal sprays and oral formulations showing potential for treating preoperative SD. Lastly, the impact of novel orexin receptor antagonists on reducing the incidence of PND by improving sleep quality merits further exploration.

### 7.2 Limitations

This article primarily focuses on the impact of insomnia disorders on PND. However, it does not address other types of SD that may also affect PND, such as obstructive sleep apnea syndrome, due to article length constraints. Additionally, the sleep intervention strategies discussed are based on the 2023 European Insomnia Guidelines and the 2017 AASM Clinical Practice Guidelines, excluding other potential interventions like music therapy, acupuncture, and herbal medicine.

## AUTHOR CONTRIBUTIONS

Chao Chen, Rui‐Xue Zhai, Xin Lan, Sheng‐Feng Yang, Si‐Jie Tang, Yu‐Xin He, Jing‐Fang Lin collected material and composed the paper; Xing‐Long Xiong and Jia‐Rong Feng assisted with language polish and figure modifications; Jing Shi and Dong‐Xu Chen modified the review.

## CONFLICT OF INTEREST STATEMENT

Dong‐Xu Chen is the editorial board members of Ibrain and a co‐author of this article, he has not been involved in the peer review process; all decisions are left to the editor‐in‐chief so as to minimize bias. The remaining authors declare no conflict of interest.

## ETHICS STATEMENT

Not applicable.

## Data Availability

Data sharing is not applicable to this article as no data sets were generated or analyzed during the current study.
